# Active Fragments from Pro- and Antiapoptotic BCL-2 Proteins Have Distinct Membrane Behavior Reflecting Their Functional Divergence

**DOI:** 10.1371/journal.pone.0009066

**Published:** 2010-02-05

**Authors:** Yannis Guillemin, Jonathan Lopez, Diana Gimenez, Gustavo Fuertes, Juan Garcia Valero, Loïc Blum, Philippe Gonzalo, Jesùs Salgado, Agnès Girard-Egrot, Abdel Aouacheria

**Affiliations:** 1 Institut de Biologie et Chimie des Protéines (IBCP), CNRS UMR5086, University of Lyon, Lyon, France; 2 Instituto de Ciencia Molecular (ICMol), Universidad de Valencia, Paterna, Valencia, España; 3 Institut de Chimie et Biochimie Moléculaires et Supramoléculaires (ICBMS), CNRS UMR5246, University of Lyon, Villeurbanne, France; 4 Departamento de Bioquímica y Biología Molecular, Universidad de Valencia, Burjassot, Valencia, España; University of Southampton, United Kingdom

## Abstract

**Background:**

The BCL-2 family of proteins includes pro- and antiapoptotic members acting by controlling the permeabilization of mitochondria. Although the association of these proteins with the outer mitochondrial membrane is crucial for their function, little is known about the characteristics of this interaction.

**Methodology/Principal Findings:**

Here, we followed a reductionist approach to clarify to what extent membrane-active regions of homologous BCL-2 family proteins contribute to their functional divergence. Using isolated mitochondria as well as model lipid Langmuir monolayers coupled with Brewster Angle Microscopy, we explored systematically and comparatively the membrane activity and membrane-peptide interactions of fragments derived from the central helical hairpin of BAX, BCL-xL and BID. The results show a connection between the differing abilities of the assayed peptide fragments to contact, insert, destabilize and porate membranes and the activity of their cognate proteins in programmed cell death.

**Conclusion/Significance:**

BCL-2 family-derived pore-forming helices thus represent structurally analogous, but functionally dissimilar membrane domains.

## Introduction

By controlling mitochondrial membrane permeability and cytochrome c release, members of the BCL-2 family are master regulators of the mitochondrial cell death pathway [1], [2], [3], [4]. BCL-2 family proteins are subdivided into three classes on the basis of their functions and the number of BCL-2 homology (BH) motifs included in their primary structure: antiapoptotic ‘multidomain’ members, such as BCL-xL, have four BH domains (BH1 to BH4), proapoptotic ‘multidomain’ members, such as BAX, possess three BH domains (BH1 to BH3), and “BH3-only” proapoptotic members, such as BID, share similarity only within the BH3 domain [4]. Most multi-BH members and several BH3-only proteins contain also a C-terminal transmembrane (TM) fragment thought to confer anchorage to mitochondrial membranes.

BCL-2 family proteins appear to regulate apoptosis by a process involving protein refolding, complex protein-protein interactions and protein-membrane interactions, current models being largely based on the functional dichotomy between opposing BCL-2-like and BAX-like effectors [1], [2], [3], [4]. Apoptosis inducers, like BAX and BAK, are thought to oligomerize and form pores in the mitochondrial outer membrane (MOM), thus causing the release of cytochrome c [5], [6], [7], [8], [9]. The proapoptotic action of BAX-like members is antagonized by prosurvival BCL-2-like proteins, which presumably carry out their protective function at the physiologically relevant locus of organelle membranes [10], [11]. Recently, BH3-only death factors have emerged as key intermediates connecting multiple noxious signals to this “dual-core” apoptotic pathway upstream of the multidomain proteins [12], [13].

Despite opposite effects on apoptosis and wide differences in amino acid sequences, three-dimensional structures and secondary structure predictions suggest that the protein fold is conserved within the ‘multidomain’ subfamily. Such a structure is composed of a group of amphipathic α-helices with a characteristic central helical hairpin [14], [15], [16], [17]. Strikingly, different from other BH3-only proteins [18], BID exhibits a 3D structure with a globular fold very similar to that of BCL-xL and BAX [19], [20]. This fold has structural analogy with that of the pore-forming subunits of several bacteriocins, such as colicin A and diphtheria toxin. Like those pore-forming toxins, BCL-2 family proteins exhibit a dual structural nature, adopting both water-soluble and membrane-bound conformations. Solution structures of prosurvival BCL-2 homologues reveal that the BH1-3 domains form a hydrophobic groove that is the docking site for BH3-only peptides and proteins. A topologically distinct BH3-binding site has recently been identified on proapoptotic BAX, which is directly involved in its functional activation as a death inducer [21]. Moreover, in cytosolic BCL-2 proteins, either proapoptotic, like BAX, or antiapoptotic, like BCL-W, BCL-xL and MCL-1, the BH3-binding pocket is obstructed by the C-terminal transmembrane (TM) domain [16], [22], [23], [24], [25], indicating a mechanistic connection between structural re-folding, BH3-binding and translocation to the outer mitochondrial membrane. Although it does not possess a TM domain, BID is also able to translocate to mitochondrial membranes upon proteolytic cleavage giving tBID [26]. This similitude is expected for homologous proteins and suggests that the conformational change of helix-bundled BCL-2 proteins is a general regulatory mechanism within the family [27], [28].

In agreement with the structural analogy with the bacterial toxins, at least four members of the BCL-2 family belonging to both the pro- (BAX, BID) and antiapoptotic (BCL-2, BCL-xL) groups have been shown to produce ion-conducting pores in model membrane systems [15]. A number of studies based on deletion and site-directed mutagenesis demonstrated that the central helices in these BCL-2 family proteins are required for pore formation and for both cytoprotection and apoptosis induction [29], [30], [31], [32], [33]. Additionally, the central helical hairpin (α5–α6) of BAX has been suggested to drive the formation of multi-spanning monomers that oligomerize to form membrane pores [34], [35], [36], [37]. The comparable helices in BID (α6–α7) have been reported to represent the minimal structural subunit required for mitochondrial targeting of a fluorescent protein [38], revealing their functional importance for membrane binding in cellular environments. Interestingly, N-terminal cleavage exposing the helices α5–α6 of BCL-xL or BCL-2 has been shown to convert these antiapoptotic members into proapoptotic products [39], [40], [41], [42], able to release cytochrome c from mitochondria [42], thus raising the possibility that the central helical region performs a similar ‘pore-forming’ function in both, pro- and antiapoptotic members.

The multiple structural and mechanistic features shared by BCL-xL, BAX and BID are in sharp contrast to their functional and sequence divergence. In the absence of detailed structural information on the active species, which are membrane bound forms, biophysical studies may provide an explanation as to how these homologous proteins, showing also a similar fold, have however opposite (BCL-xL *versus* BAX/BID) or divergent (BAX *versus* BID) functions. It has been proposed that, unlike BAX, which seemingly inserts both the central helices (α5–α6) and the tail-anchor (α9) into membranes [37], BID binds to the lipid bilayer with its central helices near parallel to the membrane surface and without significant transmembrane insertion [43], [44], [45], [46]. The situation is less clear for BCL-xL, with conflicting evidence suggesting either transmembrane insertion of α5 and α6 with a tilt of ∼40 degrees [47] or arrangement of these helices approximately parallel to the membrane surface, similar to BID [48]. These studies suggest that the central helical hairpin motif may be key to the functional differences between the various family members. This is also supported at the cellular level by the fact that BAX and BCL-2 chimeras with swapped α5 and α6 helices have reduced pro- and antiapoptotic activity, respectively, compared to their wild-type parent proteins [30]. More recently, it has been demonstrated that a chimeric BCL-xL protein containing helix α5 of BAX is converted into a proapoptotic factor [49].

Being the focus of the activity of these proteins so persistently directed toward the α5–α6 hairpin, the use of minimal systems, consisting of singular helix fragments, may help clarifying the molecular mechanisms of the full-length proteins. We have previously shown that peptides including any of the two α-helix fragments of the hairpin of BAX (α5 or α6) can independently permeabilize synthetic lipid vesicles [50], [51]. This meant that both central helices of BAX carry, by themselves, minimal structural information to insert into model lipid membranes and form pores, thereby recapitulating, at least in part, the behavior of full length BAX. In the present work, we sought to pursue this reductionist approach to clarify to what extent the ‘pore domain’ of homologous BCL-2 family proteins contributes to their functional divergence. We have used a set of synthetic peptides derived from the central helices and the α-helical BH3 domain of both antiapoptotic (BCL-xL) and proapoptotic BCL-2 family members (BAX, BID) to characterize their interaction with lipid Langmuir monolayers and their ability to disrupt membrane barrier properties using a mitochondrial cytochrome c release assay. This selection of peptides was made because, different from BAK and BCL-2, which reside in the MOM, BCL-xL, BAX, and BID are soluble cytosolic proteins that translocate to the mitochondrial membrane upon apoptotic stimuli [26], [52], [53], i.e., all three proteins exhibit similar behavior with respect to their activation and recruitment to the MOM. The results of our comparative study indicate that the central helices of BAX, BCL-xL and BID have different abilities to interact with and destabilize membranes *in vitro*, suggesting that the ‘pore-forming’ domains of these various BCL-2 family proteins have been shaped over the course of evolution to perform slightly different functions in apoptosis regulation.

## Results

### Peptide-Lipid Interaction Using Langmuir Monolayers: Choice of Systems and Control Experiments

**Figure 1:**
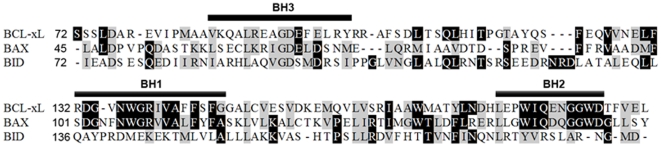
Aligned aminoacid sequences of the different BCL-2 family proteins investigated in this study. The conserved BH1-3 domains are indicated. BID shows similarity only in the BH3 region. The sequence and some general properties of the peptides analyzed are described in Table 1.

For the biophysical characterization of membrane active protein fragments, we have used Langmuir monolayers as *in vitro* model of lipid membranes. These supramolecular lipid films formed at the air-water interface are attractive membrane models [54], widely used for studying peptide-lipid interactions [55], [56], [57]. We have investigated the binding of peptides derived from the central helices of BAX, BCL-xL and BID at physiological pH with model phospholipid monolayers of a composition that imitates mitochondrial membranes. For this purpose we selected fragments encompassing the sequences corresponding to the first or second α-helices of the core hairpins of BAX, BCL-xL and BID, as defined in the water-soluble protein structures [16], [17], [19]. Because the structures of those proteins in a membrane environment is currently unavailable, for the case of the first hairpin helix of BAX and BCL-xL (α5) we analyzed both a short version (labeled “S”), including only the reported α-helical residues, and a long version (labeled “L”) that extends a few residues beyond the α-helix stretch at both ends. Divergence of the BID sequence precluded unambiguous alignment with the other two proteins and a similar peptide design. Peptides corresponding to the BH3 domain of BAX, BCL-xL and BID were also included in the study. The sequence and some general properties of the peptides analyzed in this study are described in Table 1 and Figure 1). Lipid mixtures of POPC/DOPE (2∶1) and POPC/DOPE/CL (1∶1∶1) were chosen to mimic the MOM and the contact sites between inner and outer mitochondrial membrane (MIM/MOM), respectively [58]. Penetration assays were carried out at constant area [59] by spreading the lipids at the air-buffer interface, compressing the film at π_i_ = 5 mM/m and then injecting the peptides into the subphase at a concentration of 0.2 µM (see Material and Methods). Subsequent peptide binding results typically in an increase of surface pressure. Thus, the peptide-monolayer interaction can be characterized by measurable kinetic properties, such as the initial velocity of surface pressure increase (V_i_), which informs about the affinity of the peptide for the lipid-water interface, and the final increase of surface pressure (π_max_), which can be related to the insertion ability of the peptide into the monolayer.

**Table 1:** Sequences and general physicochemical properties of the peptides used in this study.

Peptide	Sequence	Length	pI^a^	Net charge at pH 7^a^	Mean Hydrophobicity^b^	Mean Hydrophobic Moment^b^
BAX-α5S	WGRVVALFYFASKLVLKALSTK	22	10.9	4	1.14	1.97
BAX-α5L	DGNFNWGRVVALFYFASKLVLKALSTKVPELIRT	34	10.4	3	0.3	1.83
BAX-α5M	NWGRVVALFYFASKLVLKALSTKVPELIR	29	10.9	4	0.76	2.41
BAX-α5MS	NWGVVKALFYFASVLRLKALSTKVPELIR	29	10.9	4	0.76	1.06
BCLX-α5S	WGRIVAFFSFGGALSVESVDK	21	7	0	0.54	1.97
BCLX-α5L	RDGVNWGRIVAFFSFGGALSVESVDKEMQVLVSR	34	7.2	0	−0.53	1.37
BID-α6	EKEKTMLVLALLLAKKVASH	20	10.2	+2.1	−0.52	1.08
BAX-α6	ELIRTIMGWTLDFLRERLLVWIQD	24	4.6	−1	1.39	2.11
BCLX-α6	VLVSRIAAWMATYLNDHLEPWIQE	24	4.4	−1.9	0.74	2.29
BID-α7	SLLRDVFHTTVNFINQANLRTYVR	24	11	+2.1	−0.56	1.39
BAX-BH3	VPQDASTKKLSECLKRIGDELDSNMELQR	29	4.8	−1	−2.94	2.36
BAX-BH3m	VPQDASTKKLSECEKRIGNELDSNMELQR	29	4.8	−1	−3,52	1.86
BCLX-BH3	AREVIPMAAVKQALREAGDEFELRYRRAF	29	9.7	1	−1.83	1.48
BID-BH3	ESQEDIIRNIARHLAQVGDSMDRSIPPGL	29	4.6	−1.9	−1.78	3.32
BAX-α1	EQIMKTGAFLLQGFIQDRAGRW	22	10.1	1	−0.3	1.89
BCL2L10-LAAS	TARWKKWGFQPRLKEQEGDVARDSQR	26	10.8	3	−3.57	0.79

a The net charge at pH 7.0 and Iso-electric point (pI) were calculated using the Peptide property calculator (http://www.innovagen.se/).

b Mean hydrophobicity is the sum of all residue hydrophobicity indices divided by the number of residues. The mean hydrophobic moment is the vectorial sum of residue hydrophobicity indices in an Edmundson projection divided by the number of residues, assuming a projection angle of 100° (canonical α-helix). Both were calculated with the help of HydroMCalc (http://www.bbcm.univ.trieste.it/), using indices from the combined consensus hydrophobicity scale (CCS) [92].

**Figure 2:**
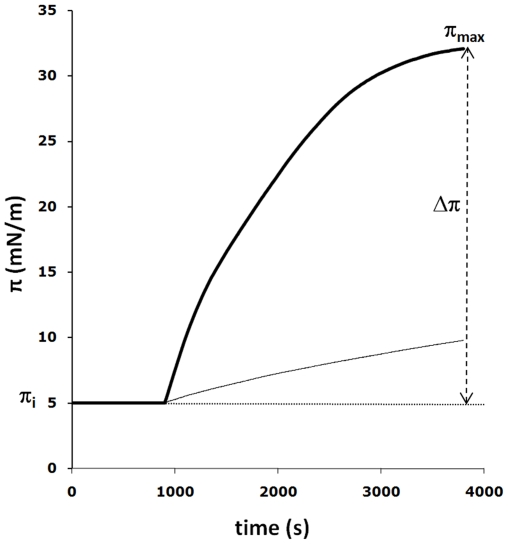
Surface pressure-time isotherms for BAX-α5S with (bold line) or without (light line) POPC/DOPE/CL lipids. The peptide was added to the subphase at a 0.2 µM concentration and the increment of π after addition of the peptide was complete in ∼1 h. Δπ was taken to be the difference between the initial surface pressure (π_i_ = 5 mN/m) and the value (π_max_) observed after the penetration of the peptide into the lipid monolayer. The initial velocity of surface pressure increase (Vi) was calculated as the slope of the curve (Δπ/Δt) at the time of peptide addition. When the peptide was injected into the subphase in the absence of a lipid monolayer, the system was allowed to stabilize for 10 min and a compression at π_i_ = 5 mN/m (the same initial surface pressure of the lipid monolayer) was applied to ensure as similar as possible interfacial conditions.

**Figure 3:**
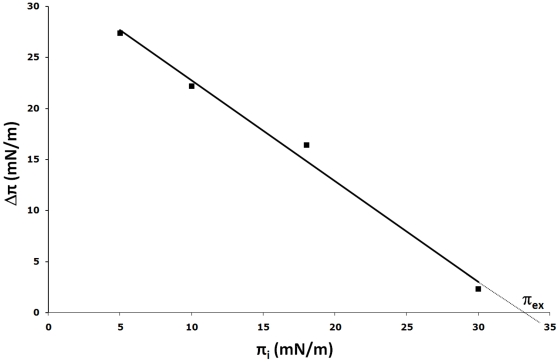
Plot of surface pressure increase *versus* initial surface pressure. Maximal surface pressure increase (Δπ) induced by injection of the Bax-α5S peptide underneath a POPC/DOPE/CL monolayer, as a function of various initial surface pressures (π_i_). The exclusion pressure (π_ex_) was determined from the abscissa intercept.

**Figure 4:**
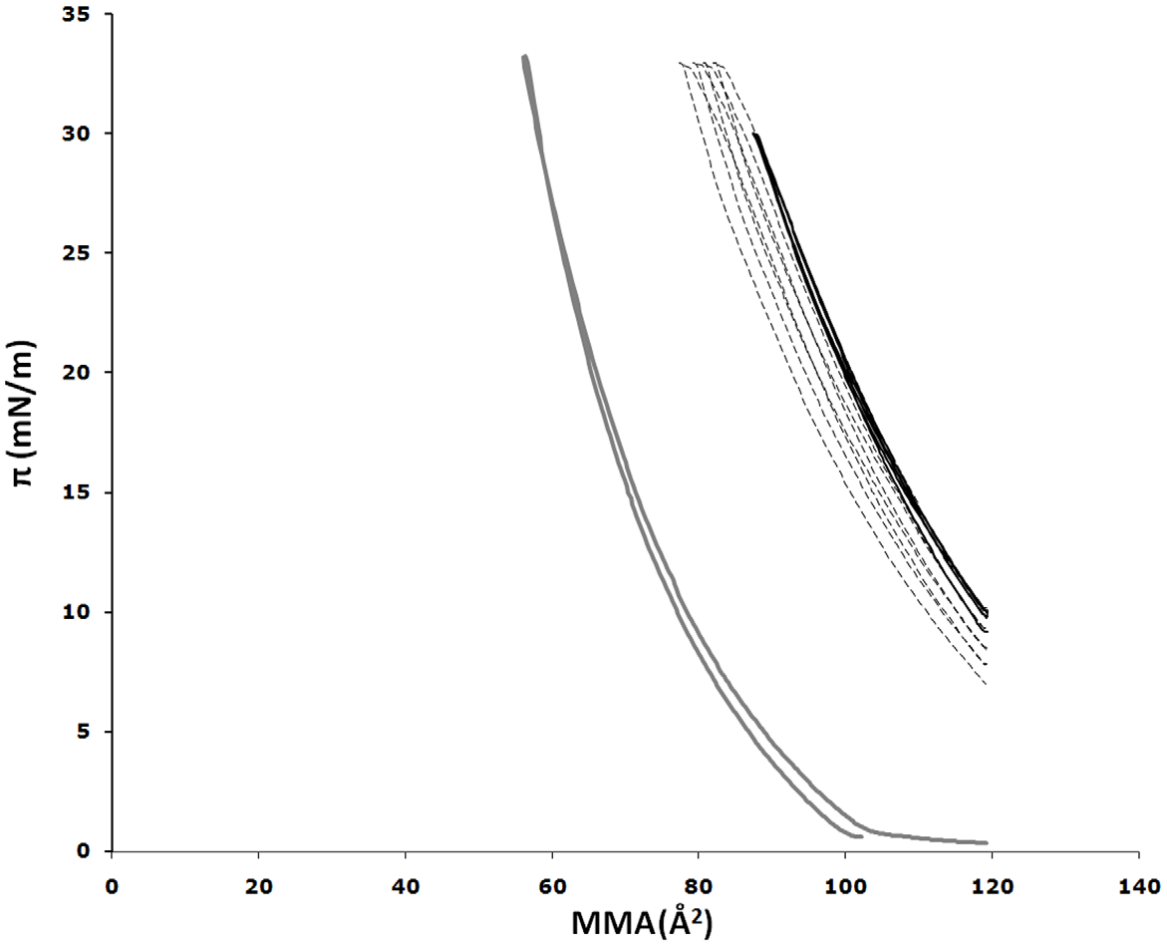
Plot of surface pressure *versus* mean molecular area (MMA). The grey lines depict the compression-decompression isotherm obtained without peptide (POPC/DOPE/CL monolayer only). The gas-like phase is present near the onset of pressure at the surface of the interface. The monolayer then changes to a liquid-expanded phase. In the presence of BAX-α5S injected at a concentration of 0.2 µM, the isotherms show a shift toward larger area (black lines), indicating peptide incorporation into the monolayer. The dashed lines were obtained after increasing the surface pressure to π>π_ex_ and indicate that the peptide was in part squeezed out from the monolayer, hysteresis being probably due to peptide ejection.

A number of control experiments were carried out in order to characterize the peptide-lipid monolayer interactions at the air-buffer interface. First, we determined surface pressure-time isotherms of Bax-α5S, a strongly amphipathic and hydrophobic peptide (Table 1), in presence or absence of lipids (POPC/DOPE/CL, see Figure 2). When the peptide was injected into a pure subphase without any lipid monolayer, the corresponding surface pressure change was Δπ = 4.8 mN/m (this value was subsequently defined as a threshold level for significant surface pressure variation). When the same injection was repeated but this time using a lipid monolayer over the aqueous subphase, at an initial surface pressure of 5 mN/m, the surface pressure rose up to Δπ = 32.1 mN/m (Figure 2). Such a level of pressure increase, which is particularly high compared to data obtained with hydrophilic proteins [60] or with BCL-2-like-derived control peptides that are not presumed to insert deeply into membranes (BAX-α1 and BCL2L10-LAAS, see Figure S1 and S2), seems unlikely to simply reflect peptide surface activity. To get further insight into the monolayer behavior in presence of peptide, we plotted the increase in surface pressure (Δπ) *versus* the initial surface pressure (π_i_) of a preformed POPC/DOPE/CL monolayer (Figure 3). From these experiments we observe an inverse correlation between the initial surface pressures and the maximum pressure increase after peptide addition, which suggests direct peptide-lipid interaction, as established for several other lipids and ligands [61]. An extrapolation of the linear relationship for Δπ = 0 gave the exclusion pressure of the peptide (π_ex_ = 33 mN/m). This latter value is above the lateral pressure reported for biological membranes (30 mN/m) [62], which indicates a high propensity of the peptide for membrane insertion [54]. Last, we plotted surface pressure–molecular area isotherms and hysteresis curves. As shown in Figure 4, the surface pressure of the lipid monolayer showed a gradual rise on compression, reaching 33 mN/m with no sign of collapse. Little hysteresis was observed in decompression after the initial compression, which is indicative of the near-equilibrium character of the isotherms. Expectedly, the monolayer was in the liquid-expanded (LE) phase at 5 mN/m, which is the initial surface pressure chosen for subsequent peptide insertion experiments. It is important to note that this phase is associated with membrane fluidity, which is a prerequisite in the study of peptide-membrane interactions. Then, we measured compression–decompression–recompression isotherms in presence of the peptide. In a first step, the lipids alone were compressed up to π_i_ = 5 mN/m. After stabilization, the peptide was injected underneath the monolayer, π_max_ was recorded (32.1 mN/m) and the monolayer was decompressed. When the monolayer was compressed again to a surface pressure π<π_ex_, greater apparent molecular areas were observed at lower surface pressures, with almost no hysteresis between the compression and decompression isotherms, which is indicative of peptide intercalation. Upon increase in surface pressure to π>π_ex_ (four decompression–compression cycles were performed), the isotherms recorded were shifted towards lower molecular areas, suggesting that the peptide component was progressively ejected from the interface. Although peptides usually show some surface activity, these results collectively suggest that (i) in our experimental conditions the observed pressure changes will mainly be due to peptide penetration into the monolayers; (ii) our approach can offer a way for accurately discriminating between peptides with weak or strong affinity for lipids or different propensity to embed themselves in the lipid monolayers.

### Insertion of BCL-2 Fragment Peptides into Phospholipid Langmuir Monolayers

**Figure 5:**
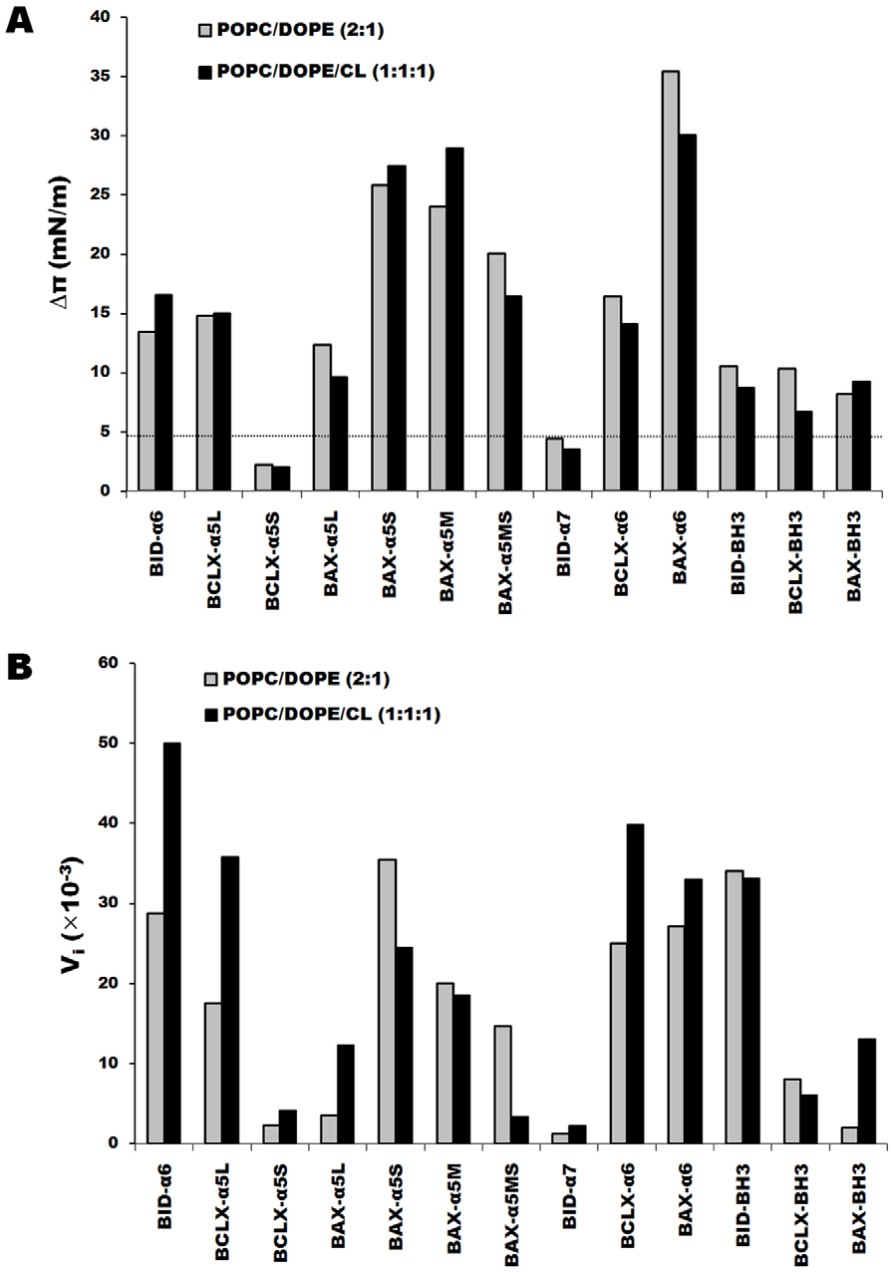
Changes in surface pressure after peptide injection. Peptides were injected underneath POPC/DOPE or POPC/DOPE/CL monolayers at constant area. *A.* Final increase of surface pressure π_max_ (mN/m) obtained for the different peptides. The dashed line denotes the threshold (Δπ = 4.8 mN/m) for significant surface pressure variation as determined using BAX-α5S without lipids (see text). *B.* Initial velocity of surface pressure increase (V_i_ = Δπ/s).

We found the highest increases in surface pressure for BAX-α6 and BAX-α5S (Figure 5), and we interpret this behavior as corresponding to a deep insertion of these peptides within the monolayers. The case of BAX-α5S contrasts with that of the longer version of the same α5 helix (BAX-α5L), for which π_max_ reaches a much lower value. Similar to the latter, we obtained low π_max_ values for the three BH3 peptides assayed (Figure 5), indicating a weak insertion in the monolayers, most likely at the level of the interface of the phospholipids. Among the rest of the peptides, BCLX-α5L, BCLX-α6 and BID-α6 showed a similar degree of π_max_, which was higher than the one of the BH3 peptides and can be interpreted as a deeper, although still interfacial, binding. Finally, BCLX-α5S and BID-α7 displayed the lowest increase of surface pressure, suggesting that these peptides were located peripherally outside the monolayers. Overall, the values of π_max_ and V_i_ correlate with each other, showing that peptide-monolayer affinity tends to be connected with the peptide insertion capacity, although with a few exceptions. For example, the affinity (V_i_) appears strong for BCLX-α5S, BCLX-α6 and BID-BH3, despite their moderate to small insertion capacity (π_max_).

Noteworthy, there are very clear differences between analogous helices from the different proteins. For the first helices of the hairpin (α5 of BAX and BCL-xL, α6 of BID) the large π_max_ value of BAX-α5S contrasts to the small value of BCLX-α5S, while BID-α6 exhibits an intermediate value. Among the second helices (α6 of BAX and BCL-xL, α7 of BID) the largest difference is between BAX-α6 and BID-α7, BCLX-α6 having an intermediate value. Together, these observations indicate that the different BCL-2 family members can be distinguished by the different degrees of insertion displayed by analogous fragments from their central helix hairpins. The two central helices of proapoptotic BAX have the highest capacity for monolayer insertion. Antiapoptotic BCL-xL displays moderate membrane insertion capacity and only for the second helix of the hairpin, and the BH3-only protein BID shows also moderate membrane insertion capacity, but this time only for the first helix of the hairpin.

With respect to the lipid composition, it had a minor effect on peptide insertion (π_max_), but for most of the peptides (especially BCLX-α5L and BID-α6) the affinities (V_i_) were higher in cardiolipin-containing model membranes.

It is interesting to see how the increase of length has an opposite effect for BAX-α5, compared to BCLX-α5, both on binding affinity and insertion efficiency. Indeed, there was a ∼5 fold increase in surface pressure with BCLX-α5L compared to BCLX-α5S, but a reduction to about half of surface pressure for BAX-α5L compared to BAX-α5S. To characterize further the effect of additional residues on membrane insertion, we assayed a medium-size BAX peptide (BAX-α5M) including the extra C-terminal hairpin turn sequence, but lacking the N-terminal flanking residues, compared to BAX-α5L (see Table 1). We observed that BAX-α5M induced changes in the surface pressure similar to those observed after injection of BAX-α5S, with a slightly smaller V_i_, indicating that it was the presence of extra N-terminal residues which caused reduction of BAX-α5L insertion. Assuming that the peptides are α-helical, a possible reason for this behavior would be a stronger amphipathicity in the case of BAX-α5M, compared to BAX-α5L, as indicated by their mean hydrophobic moment (Table 1). In fact, a weakly amphipathic version (BAX-α5MS) made by swapping the position of a pair of positively charged residues with a pair of hydrophobic residues (underlined in Table 1) shows a reduced surface pressure compared to BAX-α5M. However, these arguments have to be used with care and do not allow easy comparison between all cases (like BAX fragments against BCL-xL fragments), since the hydrophobic moment depends on the actual (unknown) structure and other factors, like the net charge or the mean hydrophobicity of the peptides may also play a role.

Collectively, these results suggest that the differences in amino acid composition and sequence between analogous α5 peptides directly influence the efficiency of monolayer binding and insertion, and that the patterns of peptide-membrane interaction are different for BAX-, BCL-xL- and BID-derived fragments.

### Structural Characterization of Peptide-Monolayer Complexes by Brewster Angle Microscopy

**Figure 6:**
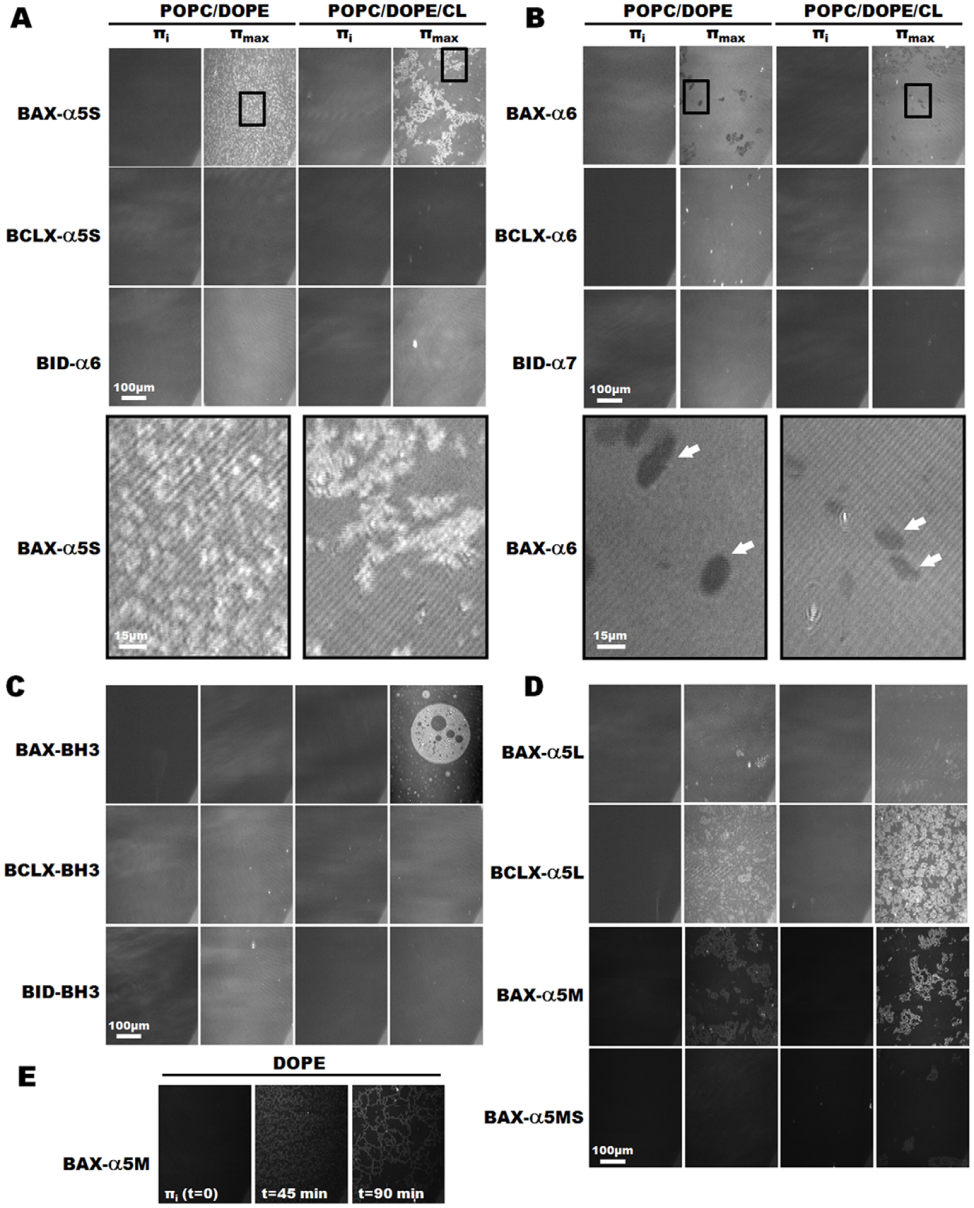
Topographic structure of the monolayers visualized by Brewster angle microscopy. BAM images were recorded at π_i_ ( = 5 mN/m) and once the plateau surface pressure (π_max_) was attained. Scale bars are included. *A.* BAM microphotographs for the first helix (BAX-α5S, BCLX-α5S, BID-α6) of the ‘pore domain’ of BAX, BCL-xL and BID. *B.* BAM images in presence of the second helix (BAX-α6, BCLX-α6, BID-α7) of the ‘pore domain’. Zoomed cutouts (low panels) are depicted for BAX-α5s (in A) and BAX-α6 (in B). *C.* BAM images in presence of the BH3 peptides. *D.* BAM images for BCLX-α5L and BAX-α5L, M and MS. *E.* BAM pictures for BAX-α5M in pure DOPE monolayers at the indicated time after peptide injection.

Next, we used Brewster Angle Microscopy (BAM) to visualize the monolayer morphology upon injection of the different peptides into the Langmuir trough subphase, underneath the compressed lipid monolayers. The BAM images were taken at an initial surface pressure of 5 mN/m (π_i_), and subsequently, when the surface pressure reached a plateau (π_max_). As shown in Figure 6, for all experiments at the initial surface pressure, the film is homogenous and with morphology typical of a Liquid Expanded (LE) phase. Addition of 0.2 µM BAX-α5S in the subphase underneath the MOM-mimicking monolayers resulted in a drastic remodeling of the phospholipid organization, with the appearance of domains of densely packed molecules (bright dots) dispersed in a background fluid phase (grey areas) (Figure 6A, first row and enlarged panels). In the MIM/MOM-like monolayers, the presence of BAX-α5S induced a more complex morphology, consisting of large regions of expanded phase (grey areas) coexisting with large irregular domains of condensed phase (bright areas). These results indicate that BAX-α5 is able to induce lipid phase segregation and most likely aggregate in spatially separated domains. Such domains appear dispersed in MOM-like monolayers but clustered in contact sites-like monolayers, probably due to the presence in the latter case of the negatively charged CL. Of note, the BAM images of MOM- and MIM/MOM-like monolayers spread at π_i_ = 30 mN/m and recorded after addition of Bax-α5S were very similar to that obtained at π_i_ = 5 mN/m (Figure S3).

In contrast, BCLX-α5S did not modify appreciably the structure of the monolayers (Figure 6A, second row), either at π_i_ = 5 mN/m or π_i_ = 30 mN/m (Figure S3), which was expected after the weak monolayer insertion observed for this peptide (see above). On the other hand, BAX-α6 induced the most striking changes in the morphology of the lipid films (Figure 6B, first row and enlarged panels), as reflected by the apparent holes observed in BAM images (marked with arrows). These round-shaped extrusions have diameters ranging from 5 to 20 µm and display sharp bright rims that correspond to an increase of local thickness at their periphery. The difference in height calculated from reflectance values was found to be 1.07±0.19 nm, between the dark area of the holes and the peripheral rims, and 0.49±0.2 nm, between the rims and the surrounding monolayer.

In presence of BID-α6 (Figure 6A, third row), the MOM-mimicking monolayer was homogenous, but a clear condensation (increase of brightness) of the monolayer could be observed, especially for the cardiolipin-containing lipid mixture. This, in agreement with the large rate of surface pressure increase (see Figure 5), suggests that BID-α6 binds efficiently to the lipid surface *via* electrostatic interactions, but without penetrating deep into the monolayer, as also suggested by the moderate π_max_ values. A similar, although less pronounced behavior is observed in the presence of BCLX-α6 (Figure 6B, second row). The monolayers displayed a homogenous surface in the presence of BID-α7 (Figure 6B, third row) or BCL-xL and BID BH3 fragments (Figure 6C, second and third row), and no brighter domains of more condensed phase were formed, in agreement with the low binding and insertion of these peptides. However, BAX-BH3, despite its weak insertion into lipid monolayers (see above), altered drastically the lipid film organization at the air-water interface in the presence of cardiolipin, forming circular domains of expanded phase trapped within larger circular domains of condensed phase (Figure 6C, first row).

The BAM images obtained with the longer versions of helix 5 from BAX and BCL-xL were in good agreement with the penetration kinetics (Figure 6D). Indeed, BCLX-α5L, which partitions more strongly than BCLX-α5S into the model monolayers, was able to change the lipid organization, as shown by the appearance of condensed domains in the BAM images (Figure 6D, second row). On the other hand, BAX-α5L, showing moderate insertion into the monolayers, did not produce appreciable lipid condensed patches (Figure 6D, first row), contrary to the short version (BAX-α5S) or the forms extended only at the C-terminal side (BAX-α5M and BAX-α5MS). Interestingly, a careful microscopic inspection of the monolayers revealed subtle differences in the way that these latter peptides change the monolayer morphology. First, in the POPC/DOPE mixture, the height of the domains differed significantly between the different cases, being: 2.54±0.31 nm, 1.99±0.24 nm, 1.04±0.13 nm, and 1.32±0.16 nm for BAX-α5S, BAX-α5M, BAX-α5MS and BCLX-α5L, respectively. Moreover, in the POPC/DOPE/CL mixture containing the BAX-α5S and BAX-α5M peptides, the condensed regions connected and formed network-like structures, a specific film topography that was not observed for the other peptides. The tendency to form networks of protruded material was also seen in monolayers of pure DOPE treated with BAX-α5M (Figure 6A and 6D), for which the domains progressively started to form small clusters to end up branching and forming a cross-linked network structure (Figure 6E).

Altogether, these data provide evidence that peptide fragments of the proapoptotic BAX protein (BH3, α5 and α6) induce important morphologic rearrangements in phospholipid monolayers. Helix α5 of BCL-xL (at least the long version) also forms phase-separated domains within the phospholipid monolayers, while BID-α6 and BCLX-α6 appear to establish superficial contacts with the monolayer interface, and no appreciable changes in the monolayer surface texture can be detected for BID-α7.

### Peptide-Induced Cytochrome c Release from Mitochondria

**Figure 7:**
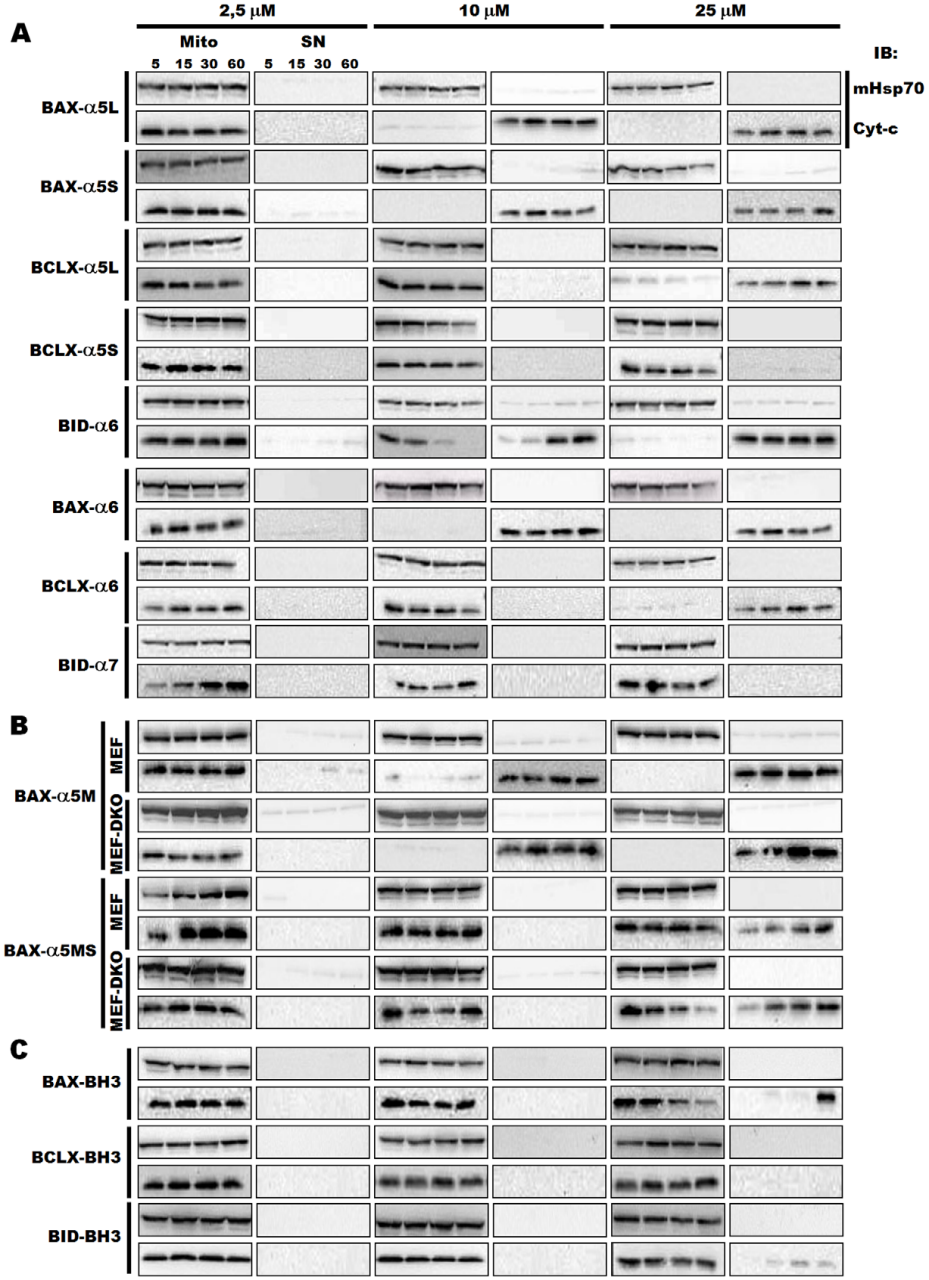
Cytochrome c release assays. Peptides were incubated with isolated mitochondria for the indicated times (min) and the release of cytochrome c was monitored by Western blotting (IB). MitoHSP70 was used as an equal-loading control for the pellet fraction. Control lanes indicate that in the preparation the MOM is intact and cytochrome c is retained within the intermembrane space. *A.* Cytochrome c release assays for the first (BAX-α5S, BCLX-α5S, BID-α6) and second helices (BAX-α6, BCLX-α6, BID-α7) of the ‘pore domain’ of BAX, BCL-xL and BID using mitochondria isolated from HEK293T cells. *B.* Cytochrome c release assays for BAX-α5M and BAX-α5MS using mitochondria isolated from MEF and MEK BAX/BAK -/- double knock-out cells (MEF DKO). *C.* Cytochrome c release assays for the BH3 peptides.

To explore the behavior of comparable fragments from BAX, BCL-xL and BID with respect to the membrane permeabilization, isolated mitochondria were used as a test system which closely resembles the *in vivo* functional context. Thus, we tested the ability of the different peptides to induce the release of mitochondrial proteins by incubating them with mitochondria isolated from HEK293T cells, and subsequently assaying the supernatant and pelleted fractions with antibodies for cytochrome c and mitoHSP70. When the different ‘pore-forming’ peptides were compared (Figure 7A), the largest release was obtained for BAX-α5S and BAX-α6, which when added at a 10 µM concentration, induced complete depletion of all mitochondrial cytochrome c after 5 min incubation. In a similar experiment with BAX-α5L, some residual cytochrome c was still present in the mitochondrial pellet after the 5 min treatment. On the other hand, incubation of mitochondria with 10 µM BID-α6 induced a slower release, with depletion of the mitochondrial pool of cytochrome c occurring only after 1 hour incubation. BCLX-α5S completely failed to release cytochrome c at all doses and times tested, whereas the BCLX-α5L and BCLX-α6 peptides induced the release of large amounts of cytochrome c, but only at the highest concentration assayed (25 µM, 5 min). These results demonstrate that peptides corresponding to the α5 and α6 fragments of BAX can porate mitochondria efficiently and independently, showing a higher cytochrome c releasing capacity than analogous peptides derived from BID and BCL-xL.

Cytochrome c release also occurred when BAX-α5M was added to mitochondria isolated from BAX/BAK double knockout mouse embryonic fibroblasts (MEF DKO), indicating that this peptide triggers MOM permeabilization in a BAX/BAK-independent manner (Figure 7B). Moreover, an increased lag time and a decreased rate of cytochrome c release were observed when mitochondria were incubated with BAX-α5MS, suggesting that amphipathicity is important for the full MOM-disrupting activity of BAX α5.

Last, we proceeded to perform similar dose-response experiments by measuring cytochrome c release from isolated mitochondria with peptides derived from the BH3 domains of BAX, BID and BCL-xL. Figure 7C shows that the BCLX-BH3 peptide induced no cytochrome c release in this mitochondrion-based system. On the other hand, the BH3 peptides from BAX and BID triggered mitochondrial cytochrome c release, although with markedly less efficiency (starting at 25 µM after 15–30 min of peptide exposure) than BAX-α5, BAX-α6 or BID-α6. However, treatment of mitochondria with a BAX BH3 mutant (BAX-BH3m), harboring substitutions (L^63^E/D^68^N) predicted to disrupt the interaction with antiapoptotic BCL-2-like proteins, failed to induce cytochrome c release even at 100 µM (data not shown). This result indicates that the observed activity, at least of the BAX-BH3 peptide, is primarily due to inactivation of endogenous prosurvival BCL-2 family proteins.

In conclusion, there was a good agreement between the rates of cytochrome c release from isolated mitochondria and the effects observed in the Langmuir monolayer and BAM studies. Our data illustrate a particularly strong capacity of the BAX central helices to trigger MOM permeabilization, compared to analogous segments in BID and BCL-xL or to isolated BH3 peptides.

## Discussion

The active forms of BCL-2 family proteins refold and work in lipid membranes through a complex mechanism that has started to be disentangled only recently [10], [11]. However, the inherent difficulties for the structural study of protein-membrane complexes still hinder the appearance of specific molecular models of the active species. For example, although the sequence of events for the tBID/BAX driven mitochondrial poration process, as well as the general role of these two proteins, appear now clear [10], the details of tBID-BAX binding and refolding in the membrane, BAX oligomerization and pore formation are still to be discovered. Similarly, although the inhibitory mechanism exerted by BCL-xL seems now well defined, as an effective blocker of the tBID docking site for BAX, as well as of BAX oligomerization [11], the way these inhibitory actions are performed at a structural level is unknown. Given the clear connections between the three BCL-2 subfamilies, which can be singularized at specific active domains, and given the protagonism of the lipid membrane for their activation mechanism, a thorough comparative study of membrane activity and membrane interactions of analogous fragments from the three types of proteins is key to understand the bases of their different behavior. For this study, we have synthesized a set of peptides with the sequences of the membrano-tropic core hairpin helices plus the well conserved BH3 domains found in the three homologous proteins BCL-xL, BAX and BID. This choice was made because, different from BAK and BCL-2, which reside in the MOM, BCL-xL, BAX and BID all normally exist as soluble proteins in the cytosol, from where they translocate to mitochondrial membranes upon apoptotic stimuli [26], [52], [53], i.e., they exhibit similar behavior with respect to their activation and recruitment to the MOM.

The interaction of the peptides with lipid membranes and the remodeling of the membrane organization were investigated here using Langmuir monolayers. This system, employed extensively for the study of amphipathic and antimicrobial peptides [55], [56], represents an interesting model of actual membrane surfaces due to the absence of intrinsic curvature. We used a monolayer composition mimicking that of MOM and MOM/MIM regions, and we characterized the insertion of the BCL-2-derived peptides by monitoring the time-dependent changes of surface pressure at a constant film area. The intrinsic membrane interaction capacity of single and double helix fragments from the same proteins has been investigated before [46] using bioinformatics and glycosylation mapping methods. However, the work presented here is quantitative and allows a better comparison of the behavior of the different systems. Additionally, we performed a parallel comparison of peptide effects on monolayer properties, and because the peptide-lipid monolayer interactions measured under our experimental conditions might not be fully representative of protein-membrane complexes, we conducted additional *in vitro* tests to compare the poration activity of the different peptide versions directly on mitochondrial membranes.

We found a higher penetration in lipid monolayers of the central helices (α5 and α6) of BAX, compared to those of BCL-xL and BID (α6 and α7). Thus, while both individual hairpin fragments of BAX penetrated rapidly and deeply into the monolayers, in the cases of BCL-xL and BID, the membrane interaction parameters are overall smaller, especially for helices BCLX-α5S and BID-α7. In the previous work [46], the first helices of the hairpins from the three proteins were found to be able to insert in a TM fashion, while the second helices inserted only in the presence of the first (as a complete hairpin). This different behavior compared to the one described here may be due to the protein context (chimera of the model membrane protein Lep), the different membrane system (endoplasmic reticulum membranes) and (or) to a role of the translocon machinery used in the former assays [46].

In principle, it is reasonable to assume that the observed differences in peptide behavior depend mainly on their hydrophobicity and charge properties. However, such relationships should be made with care, since the peptide-membrane interactions depend also on other factors, like amphipathicity (hydrophobic moment) which are a function of the unknown peptide structure. For example, BAX-α5S and BAX-α6, showing the highest hydrophobic character (Table 1), also exhibit the strongest tendency to interact with lipids. Such a membrane insertion capacity correlates well with the net positive charge of BAX-α5S, although it appears contradictory, at least for the binding with negatively charged CL-containing monolayers, with the negative charge of BAX-α6. Nevertheless, the reduction of both the hydrophobicity and positive charge of the longer version of this helix, BAX-α5L, as well as the (partial) recovery of both factors in the BAX-α5M version, would explain the smaller tendency to interact with the monolayers of the first one, and the recovery of the membrane insertion capacity of the second. Additionally, such behavior of the different BAX versions is also in agreement with their expected hydrophobic moment, if the peptides are assumed α-helical. We can also expect that the hairpin made of the two fragments (BAX-α5α6) would have an enhanced membrane insertion capacity, as suggested earlier by glycosylation mapping experiments [46]. Based on similar grounds, we can roughly explain the weak to moderate monolayer insertion of the BH3 peptides as well as BID-α6, BID-α7 and BCLX-α5L, all weakly hydrophobic. However, a detailed comparison, case by case, cannot easily be made without knowledge of the structure. For instance, the BH3 peptides are more polar, but exhibit a stronger binding to monolayers, than BID-α7. Also, the weak membrane binding and insertion of BCLX-α5S compared to BCLX-α5L appears difficult to justify since the first is more hydrophobic than the second (Table 1).

A way to evaluate further the peptide-monolayer interactions is by investigating the structural reorganization of the lipid monolayers as observed in BAM images. Brighter domains were clearly apparent following binding to the monolayers of the different versions of BAX-α5 and for BCLX-α5L. In both cases, these discrete domains may be considered as evidence of nucleation of 3D structures, which we interpret as peptide aggregates interacting with phospholipids. The BAM images taken after BAX-α5S injection underneath pure DOPE or POPC/DOPE/CL monolayers featured cross-linked domains, suggesting long-range interactions among the peptidolipidic aggregates. Importantly, the aggregation of BAX-α5S might be related to an intrinsic propensity of the peptide to oligomerize, which is clearly not the case for BCLX-α5S. In comparison, BCLX-α6 and the central helices of BID have no tendency to aggregate and form phase-separated domains. Rather, BCLX-α6 in POPC/DOPE and BID-α6 in POPC/DOPE/CL produced a homogeneous increase of monolayer thickness without significant surface remodeling, suggesting peptide adsorption at the monolayer surface. Noteworthy, BID-α6 binding was found to induce formation of an LC phase characterized by a close packing and rigid arrangement of the lipids that probably have an almost vertical orientation.

BAX-α6 modified the surface organization of the phospholipid monolayers by formation of holes separated from the surrounding lipid phase by a belt of increased thickness. This film heterogeneity could be attributed to a 2D-3D reorganization of BAX-α6 at the interface, the peptide first attaching to the monolayer through its hydrophobic residues, which are sparsely spaced along its length, and then recruiting lipids to create lipid-poor domains.

Last, BAX-BH3 induced circular domains of two co-existing phases, represented by bright and dark regions in monolayers with CL. These BAM images may reflect an abundance of peptide domains (bright) on a phospholipid background (dark), with some lipid domains (dark) electrostatically trapped within the peptide domains. Noteworthy, this unexpected behavior was observed only for the BH3 domain of BAX, and may indicate a role in membrane binding specifically for this protein (see below).

Strikingly, our experiments show a close relationship between the behavior of the different peptides in monolayers with MOM- and MOM/MIM-like composition and their cytochrome c releasing activity from mitochondria. Thus, peptides promoting the formation of condensed domains (as observed in BAM images); i.e., the different BAX-α5 peptides (and to a smaller extent BCLX-α5L), are seen to induce cytochrome c release from mitochondria in a BAX/BAK-independent manner. We can then conclude that the interaction of these peptides with the surface of the outside leaflet of the MOM can promote the formation of pores, probably of lipidic nature [50], [51], [63], [64], leading to cytochrome c leakage. Moreover, the concentration dependence of the MOM-disrupting activity may indicate in-membrane oligomerization of these peptides, which would be consistent with their ability to phase separate and form condensed aggregates. BAX-α6 affected also drastically the monolayer organization, forming large holes that may relate to a capacity to produce pores. BID-α6 and BCLX-α6 altered the surface properties of the monolayer by binding to the interface of phospholipids. Thus, these latter peptides could destabilize membranes in a way similar to that described for antibiotic peptides [65], [66], [67]. The lack of effect of BID-α7, BCLX-α5S and BCLX-BH3 on isolated mitochondria is in good agreement with their weak interactions with the monolayer surface and their low ability to partition into membranes. On the other hand, the case of BAX-BH3, showing induction of cytochrome c release, is special, since mutations at residues required for heterodimerization with prosurvival BCL-2-like proteins abolished such an activity. It is thus likely that this peptide acts primarily through neutralization of antiapoptotic BCL-2 proteins which are present in the *in vitro* mitochondrion-based system.

Therefore, as a whole, the differences in effects observed with isolated mitochondria generally correlate with the peptide capacity to penetrate into the monolayer surface or to affect its physical structure. However, it also appears that mitochondrial membrane poration can be associated with more than one pattern of peptide-membrane interaction.

One unexpected finding of this study is that the isolated BH3 peptide from BAX had a significant effect on the morphology of the CL-containing monolayers, although this does not seem to be accompanied by an intrinsic capacity to form pores in mitochondrial membranes (see above). Interestingly, BAX-BH3, if assumed α-helical, would be strongly amphipathic (Table 1) and thus would be expected to interact at the interface of lipid membranes. Additionally, it exhibits a KKLSE sequence that could approximate to several cardiolipin-binding motifs found in other proteins [68], [69]. This altogether suggests an active role of the BH3 helix for the interaction of full length BAX with membranes, and more specifically at contact sites between the inner and outer mitochondrial membranes (where CL is present). A localized destabilization of the membrane structure, induced by the interaction of BAX-BH3 with CL, may lower the energetic cost for inserting other amphipathic segments of BAX into the lipid matrix of the membrane. In agreement with this idea, CL has been reported to drive BAX insertion into synthetic liposomes and mitochondria and to enhance large pore formation *in vitro* [6], [70], [71], [72]. Although such effects cannot be easily assessed using single BAX peptides, our results suggest a new role for the BH3 domain of BAX as a contributing membrane-binding region, apart from its well documented role on interprotein interactions.

Although our reductionist study cannot mirror all the complexity of the *in vivo* natural systems, it shows that peptide fragments derived from the central helical hairpins of the three prototype proteins BAX, BCL-xL and BID have distinct innate capacity to bind and to insert into lipid monolayers, to induce physical changes in the monolayer state, and to trigger cytochrome c release from isolated mitochondria. Moreover, such a differentiated physicochemical behavior correlates well with the cellular function of these proteins.

Integrating previous literature, our experimental data fit into a model where (i) the BID central helices establish interfacial contact with the mitochondrial membrane surface [43], [44], [45], [46], helix α6 of BID assists BAX to permeabilize membranes by changing the material elastic properties of the lipid bilayer surface; (ii) the BAX central helices have the highest capacity to interact with mitochondrial membranes at physiological pH [51], [71], [73], [74], to insert deeply into the membrane and to homo-oligomerize, thereby creating pores that allow for cytochrome c release; (ii) BCL-xL central helices have the ability to bind to lipids [42], [74], [75], insert within mitochondrial membranes [47] but with much lower affinity/efficiency than BAX α5-α6 [42], [48], [76], and with much reduced tendency to oligomerize and porate membranes. When tentatively extrapolated to the full length proteins, this model predicts that BID is not by itself a crucial component of the MOM permeabilization machinery, but functions as a BAX activator, by favouring BAX docking in the membrane [10], [11] and pore formation [6], [71], [77], [78], [79], and through binding *via* its BH3 domain to antiapoptotic members of the BCL-2 family. In contrast to BID, membrane binding of activated BAX is followed by a membrane integration step, in which the central helices play a crucial role in oligomerization and MOM permeabilization [37]. The BCL-xL central helices, while still competent for membrane binding and insertion, are not self-assembling into pores at physiological pH [49] but, rather, function as chain terminators of nascent BAX α5–α6 pore-forming polymers [10]. Because the BCL-xL and BID central helices conserve residual membrane-disrupting activity [42], [46], [80], [81], [82], fine-tuned activation mechanisms have evolved to protect mitochondrial membranes from their spontaneous insertion.

In conclusion, by providing quantitative estimates for the interaction of BCL-2 family-derived peptides with membranes, our study gives important insights as to how (evolutionary) homologous and structurally analogous BCL-2 family proteins are functionally dissimilar. We propose that the central helices forming the so-called ‘pore domain’ of BAX, BCL-xL and BID, as well as the helix corresponding to domain BH3, which were shown here to exhibit distinct membrane behavior *in vitro*, are directly involved in the functional divergence of these proteins. Because the assayed peptides exhibited membrane activity to a similar degree in isolated mitochondria and lipid monolayers, our reductionist system can be particularly useful for the identification of key residues that determine functional differences between BCL-2 protein subfamilies.

## Materials and Methods

### Chemicals

Lipids were purchased from Avanti Polar Lipids Inc. Solvents (chloroform, methanol, ethanol and dichloromethane) are of analytical grade and used without further purification. Each lipid was diluted in chloroform/methanol (9∶1, v/v) at a final concentration of 1 mM. Mixed solutions of POPC/DOPE (2∶1 molar ratio) and POPC/DOPE/CL (1∶1∶1 molar ratio), prepared extemporaneously, were used as spreading solutions. Ultrapure water (resistivity = 18.2 MΩ•cm) obtained from a Millipore four-cartridge purification system (Millipore, France) was employed to prepare peptide solutions and buffer subphases (Hepes buffer, pH 7.4, GIBCO).

### Peptides

BAX-α5M, BAX-α5MS, BAX-BH3, BAX-BH3m, BCLX-BH3, BID-BH3, BCL2L10-LAAS peptides were purchased from GeneCust EUROPE at a 2 or 5 mg scale and were delivered with >95% purity (HPLC). BAX-α5S, BAX-α5L, BCLX-α5S, BCLX-α5L, BID-α6, BAX-α6, BCLX-α6, BID-α7 and BAX-α1 were prepared by solid-phase synthesis as reported [50] in an Applied Biosystems ABI 433A Peptide synthesizer (Foster City, CA, USA) using Fmoc chemistry and Tentagel S-RAM resin (Rapp Polymere, Tübingen, Germany; 0.24 mEq/g substitution) as a solid support. Peptides were purified using a C18 semi-preparative reversed-phase column (Merck, Darmstadt, Germany) by HPLC, to a >95% purity, and their identity was confirmed by Mass Spectrometry. Peptide concentrations were determined from UV spectra using a Jasco spectrophotometer (Jasco, Tokyo, Japan).

### Interfacial Film Formation and Surface Pressure Measurements

Monolayer experiments were performed on a computer-controlled Langmuir film balance (KSV 2000, three multi-compartment system, KSV Instrument Ltd., Finland) working in a symmetrical compression mode and enclosed in an opaque cabinet. The rectangular trough (V = 85 mL ±1 mL, S = 119.25 cm^2^) was made of Teflon and the mobile barriers were made in Delrin. The surface pressure, π defined as γ_0_ – γ, where γ_0_ is the surface tension of the pure aqueous subphase and γ the surface tension exerted by phospholipids at the subphase surface, was measured by the Wilhelmy's method using a platinum plate with an accuracy of ±0.05 mN/m. The trough was filled with Hepes buffer (pH 7.4), used as subphase and thermostated with a water circulating bath (Lauda E100, LAUDA France, SARL). In all experiments, the subphase temperature was maintained at a constant value of 22.5±0.5°C.

Phospholipid monolayers were formed by deposition of 14 µl of spreading solutions at a clean air/buffer interface by means of a micropipette to reach a final amount of 14 nmol of phospholipids. After complete evaporation of the solvent (∼15 min), the monolayer was slowly compressed up to a defined lateral pressure (initial surface pressure π_i_) at a rate of 0.045 nm^2^ molecule^−1^ min^−1^. The value of π_i_ = 5 mN/m was chosen because it lies between the surface pressure set-off and surface pressures (e.g. 30 mN/m, which corresponds to a tightly packed film) where many peptides of the study will not insert. Moreover, initial precompression of the monolayers at 5 mN/m does form well-behaved, cohesive monolayer films at the interface, and therefore minimizes the chaotic association of peptides with disorganized lipids and the likelihood of opportunistic behavior [60]. A 10 minute lag time was necessary for the monolayer relaxation and to check the monolayer stability at fixed constant surface pressure.

Peptide interaction was then investigated after injection of a defined volume of stock peptide solution under the compressed phospholipid monolayer in the buffer subphase gently stirred with a magnetic bar. The injection was performed with a Hamilton microsyringe at a constant area. The peptides were injected under the lipid monolayers and not deposited over the monolayer surface in order to minimize the effects due to the surfactant properties of peptides [83], [84].

The kinetics of surface pressure evolution due to subsequent peptide interaction with the monolayer was recorded. The final increase of surface pressure (π_max_) and the initial velocity of surface pressure increase (V_i_) were determined from the kinetics curves. Each injection was performed independently in duplicate with a fresh film and subphase (see Figure S1 for raw data).

### Brewster Angle Microscopy Experiments

The morphology of monolayers at the air/water interface, before and after peptide interaction, was observed by Brewster Angle Microscopy [85]. This technique uses the zero reflectance of an air/water surface for parallel polarized light at the Brewster angle of incidence (53° for the air/water interface). The different phases of a monolayer lead to a measurable change in reflectivity, thus allowing the visualization of monolayer morphology. The Brewster Angle Microscope (EP^3^-SW, Nanofilm, Germany) mounted on the Langmuir trough was equipped with a laser (532 nm, 50 mW), a polarizer, an analyser and a CCD camera with a x10 magnification lens. The Brewster Angle Microscopy (BAM) images coded in gray level were recorded with CCD scanning camera, using proprietary motor control circuitry with completely hands-off computer-controlled system. The spatial lateral resolution of the microscope was about 2 µm and the image size was 493×383 µm. The calibration procedure of the BAM software was used to evaluate the thickness of the layer at the interface. This procedure allows determining the linear function between the gray level of the images and the reflectance of the sample as reported in [86]. From the reflectance value, and knowledge of experimental Brewster Angle and optical index of the film, the thickness of the layer at the interface is determined using the BAM thickness model [87]. Additionally, with Brewster angle microscopy, information on the fluidity of the film can be obtained, by observing the geometry of the domains at the water surface. Thus, the condensed phase appears brilliant as compared to the liquid expanded phase, which shows as a dark background.

### Cytochrome c Release Assays

Crude mitochondria were prepared from HEK 293T, mouse embryo fibroblast (MEF) or BAX/BAK double knockout MEF cells (MEF-DKO). In brief, cells were mechanically broken using a 2 ml glass/glass Dounce homogenizer (Kontes) (30 strokes). Homogenates were cleared at 1500 g and mitochondria were spun down at 10 000 g. For cytochrome c release assays, 30 µg of crude mitochondria were resuspended at 1 mg/ml in KCl buffer supplemented with succinate (5 mM) and EGTA (0.5 mM). Peptides (2.5, 10 or 25 µM) were added to the samples and incubations were carried out at 30°C under agitation (300 rpm). At the indicated time points, samples were centrifuged (5 min, 10 000 g, 4°C); supernatants and pellets were recovered and analyzed by immunoblotting for Cytochrome c and mitoHsp70.

## Supporting Information

Figure S1 Plots of surface pressure versus time for the different peptides used in the study. Records #1 and #2 refer to duplicate experiments, carried out using a fresh film and subphase. The different steps of the experiment are shown across the second plot (BAX-α1 POPC/DOPE/CL). Lipids used for forming the monolayer are first deposited (d; the arrow marks t = 0). The monolayer can then be compressed (c) and allowed to equilibrate for ∼10 min (r, relaxation). A desired volume of peptide solution is injected below the monolayer (inj; arrow indicates time of injection) and surface pressure variations (SPV) are recorded as a function of time.
(0.67 MB PDF)

Figure S2 BAM images for control peptides BAX-α1 and BCL2L10-LAAS with POPC/DOPE or POPC/DOPE/CL lipids. See Figure S3 for surface pressure-time isotherms. BAX-α1 (of sequence EQIMKTGAFLLQGFIQDRAGRW) corresponds to the first helix localized at the N-terminus of BAX. BCL2L10-LAAS corresponds to the connecting region between predicted α5-α6 helices of BCL2L10, a prosurvival member of the BCL-2 family. Sequence of this interhelical segment (termed LAAS for Long Amino Acid Stretch, Zhang et al. 2001) is as follows: TARWKKWGFQPRLKEQEGDVARDSQR. Although BAX-α1 has been proposed to serve as a mitochondrial addressing sequence [88], [89], recent data demonstrated that this segment is a non-membrane active regulatory motif [90], [91]. The structural turn between the predicted α5 and α6 helices harbors a dozen additional residues in the human BCL2L10 protein which are not present in other BCL-2 family members. Both BAX-α1 and BCL2L10-LAAS are predicted to bind the surface of the lipid membrane but are not presumed to drive the membrane penetration of the whole proteins. Results indicate that both peptides have very weak interaction with the lipid monolayers (Δπ = 4 and 3.4 for BAX-α1 and Δπ = 3.4 and 5.5 for BCL2L10-LAAS in POPC/DOPE and POPC/DOPE/CL monolayers, respectively) and do not affect monolayer structure. See legends to Fig. 5 and Fig. 6 for experimental details.
(7.53 MB TIF)

Figure S3 BAM images acquired at initial surface pressures of 5 mN/m or 30 mN/m for BAX-α5S and BCLX-α5S. The BAM images of MOM and MIM/MOM-like lipid monolayers were recorded before (right) and after (left) addition of 0.2 µM BAX-α5S or BCLX-α5S into the subphase.
(10.20 MB TIF)
