# Supplementary figures and images for: Active Fragments from Pro- and Antiapoptotic BCL-2 Proteins Have Distinct Membrane Behavior Reflecting Their Functional Divergence
Source: PLoS One. 2010 Feb 5;5(2):e9066. doi: 10.1371/journal.pone.0009066 (PMC2816717; doi:10.1371/journal.pone.0009066)

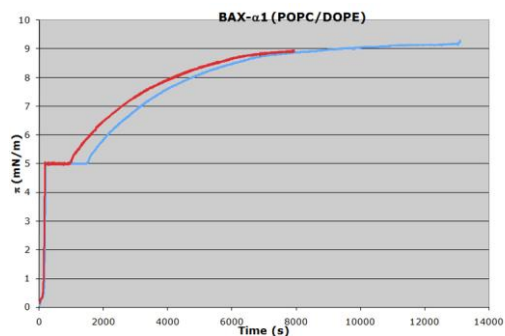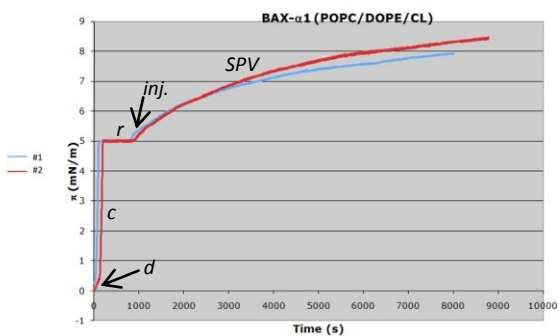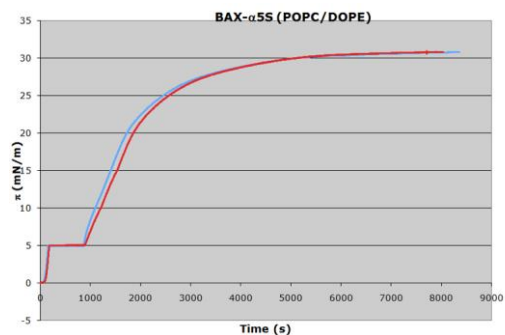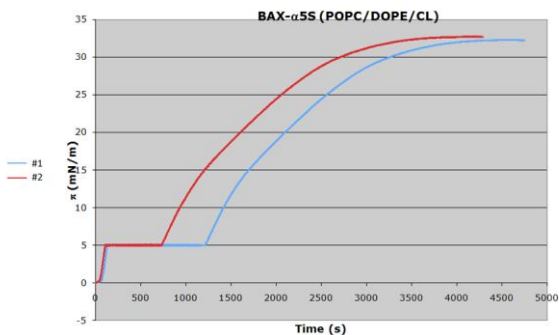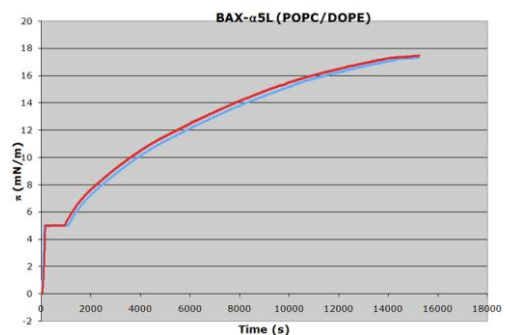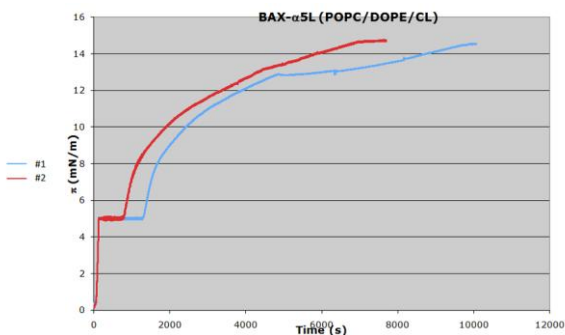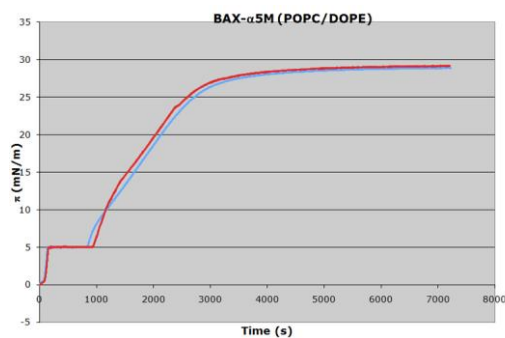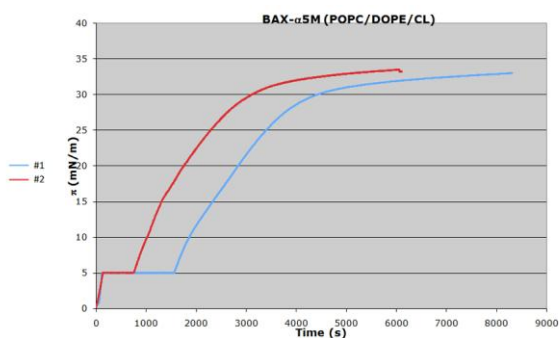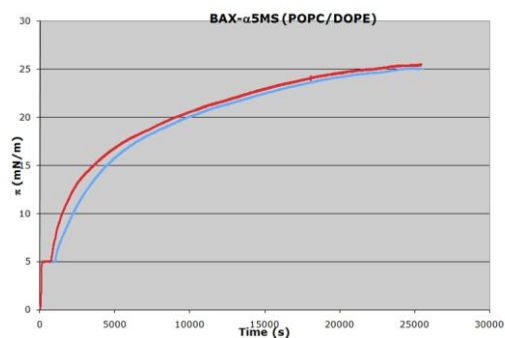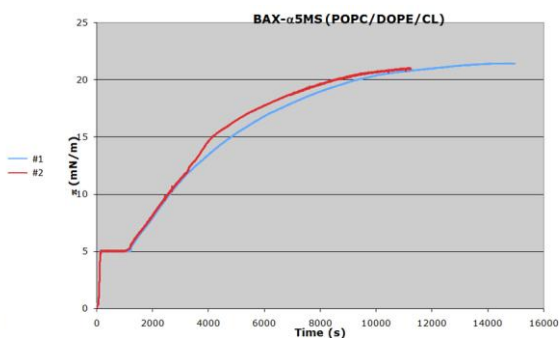

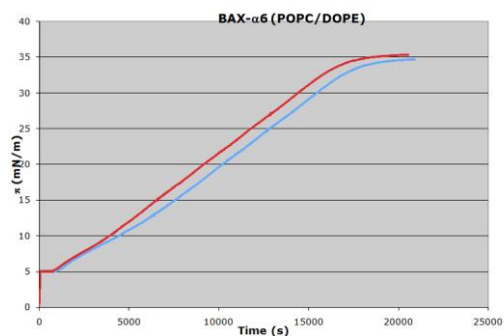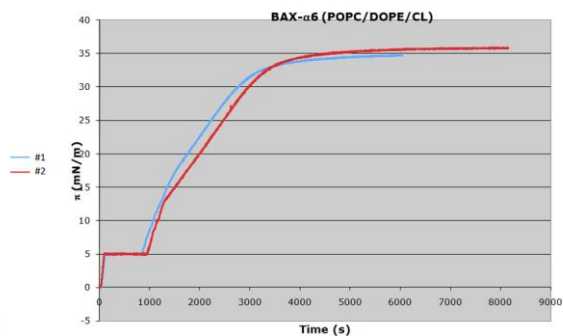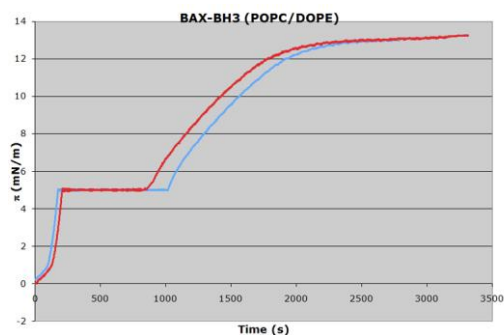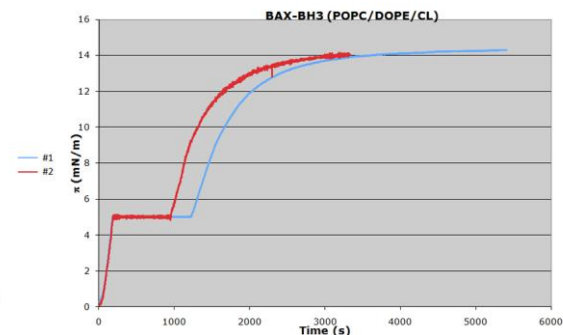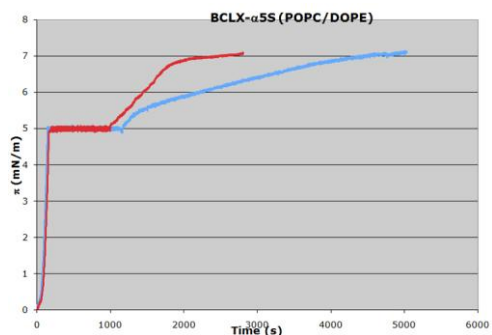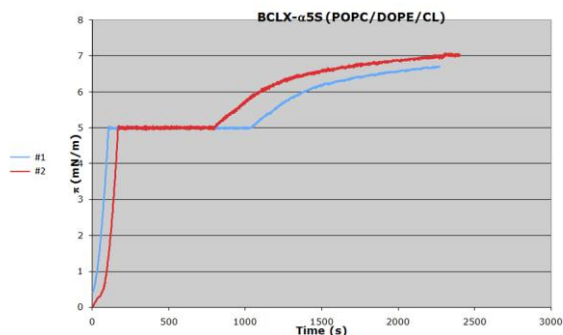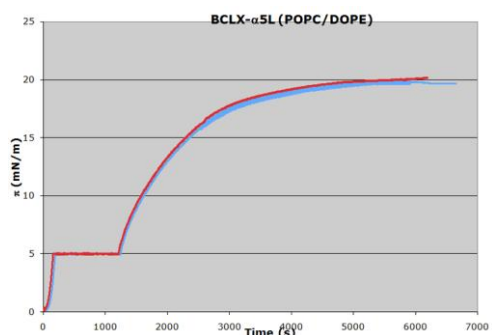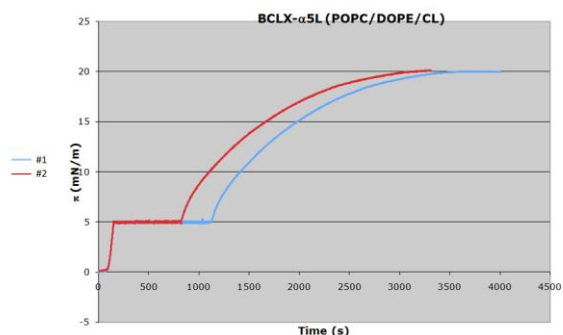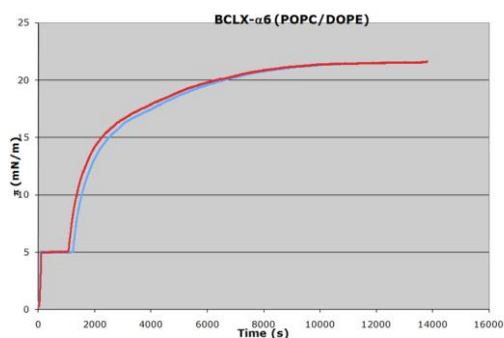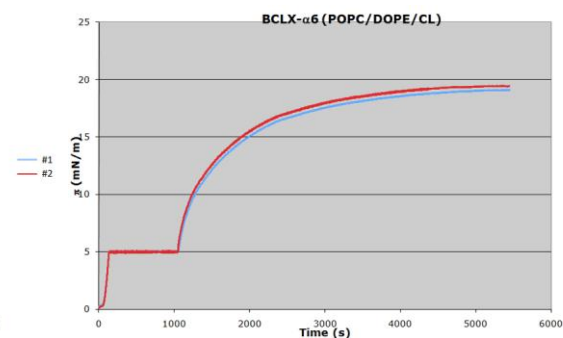

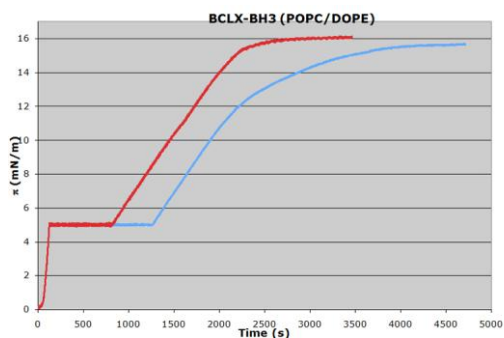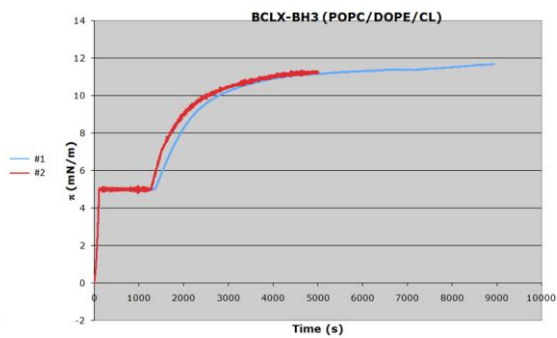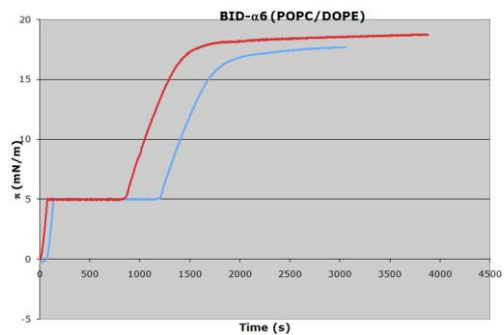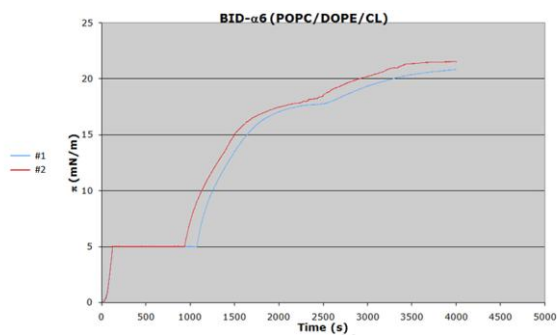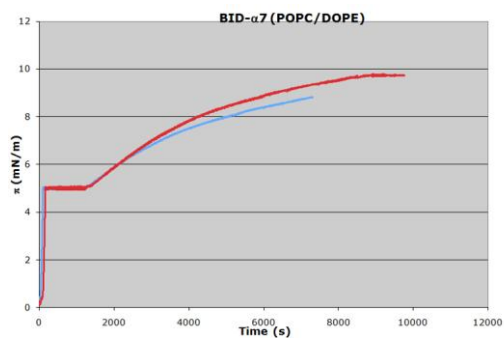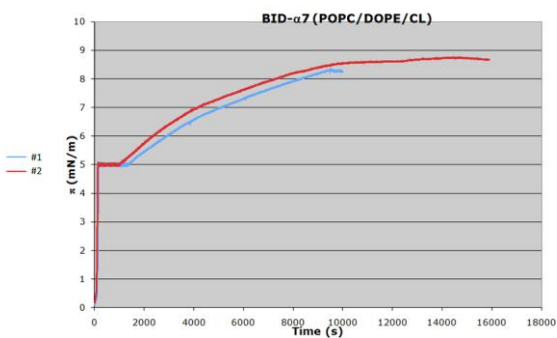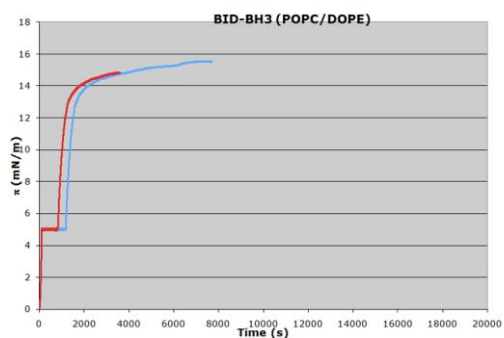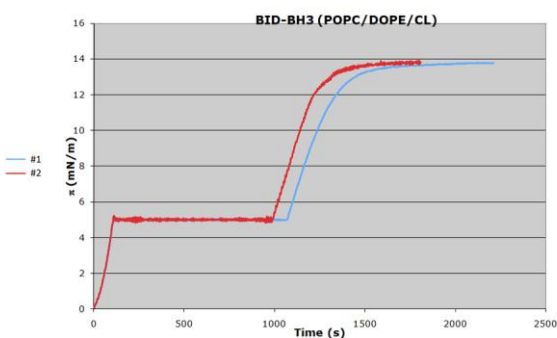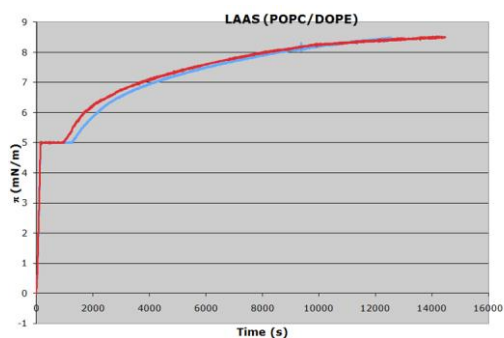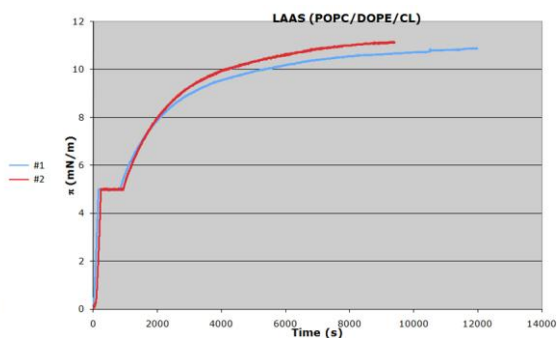

Supplement: Figure S1 — Plots of surface pressure versus time for the different peptides used in the study. Records #1 and #2 refer to duplicate experiments, carried out using a fresh film and subphase. The different steps of the experiment are shown across the second plot (BAX-α1 POPC/DOPE/CL). Lipids used for forming the monolayer are first deposited (d; the arrow marks t = 0). The monolayer can then be compressed (c) and allowed to equilibrate for ∼10 min (r, relaxation). A desired volume of peptide solution is injected below the monolayer (inj; arrow indicates time of injection) and surface pressure variations (SPV) are recorded as a function of time. (0.67 MB PDF) [file pone.0009066.s001.pdf]

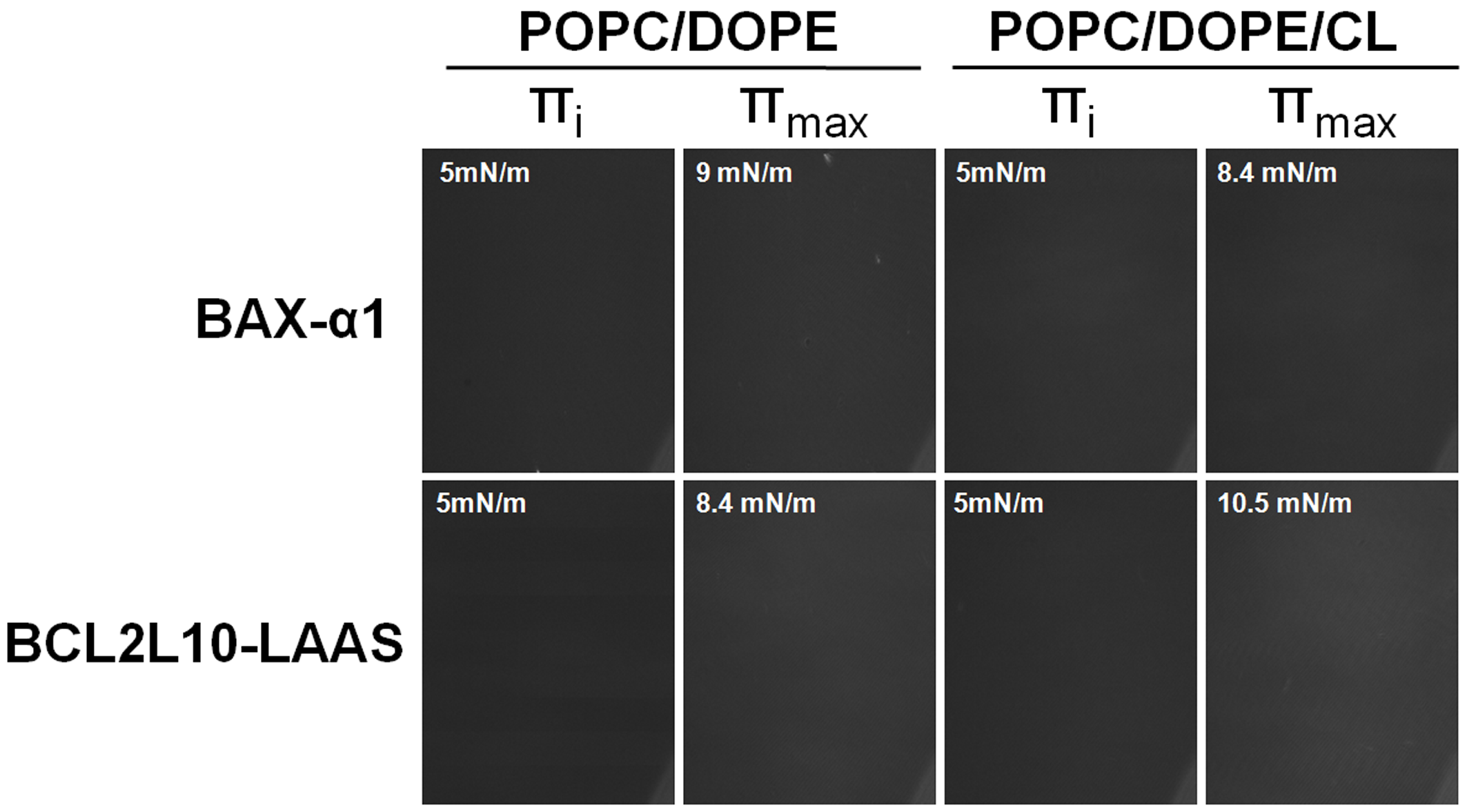

Supplement: Figure S2 — BAM images for control peptides BAX-α1 and BCL2L10-LAAS with POPC/DOPE or POPC/DOPE/CL lipids. See Figure S3 for surface pressure-time isotherms. BAX-α1 (of sequence EQIMKTGAFLLQGFIQDRAGRW) corresponds to the first helix localized at the N-terminus of BAX. BCL2L10-LAAS corresponds to the connecting region between predicted α5-α6 helices of BCL2L10, a prosurvival member of the BCL-2 family. Sequence of this interhelical segment (termed LAAS for Long Amino Acid Stretch, Zhang et al. 2001) is as follows: TARWKKWGFQPRLKEQEGDVARDSQR. Although BAX-α1 has been proposed to serve as a mitochondrial addressing sequence [88], [89], recent data demonstrated that this segment is a non-membrane active regulatory motif [90], [91]. The structural turn between the predicted α5 and α6 helices harbors a dozen additional residues in the human BCL2L10 protein which are not present in other BCL-2 family members. Both BAX-α1 and BCL2L10-LAAS are predicted to bind the surface of the lipid membrane but are not presumed to drive the membrane penetration of the whole proteins. Results indicate that both peptides have very weak interaction with the lipid monolayers (Δπ = 4 and 3.4 for BAX-α1 and Δπ = 3.4 and 5.5 for BCL2L10-LAAS in POPC/DOPE and POPC/DOPE/CL monolayers, respectively) and do not affect monolayer structure. See legends to Fig. 5 and Fig. 6 for experimental details. (7.53 MB TIF) [file pone.0009066.s002.tif]

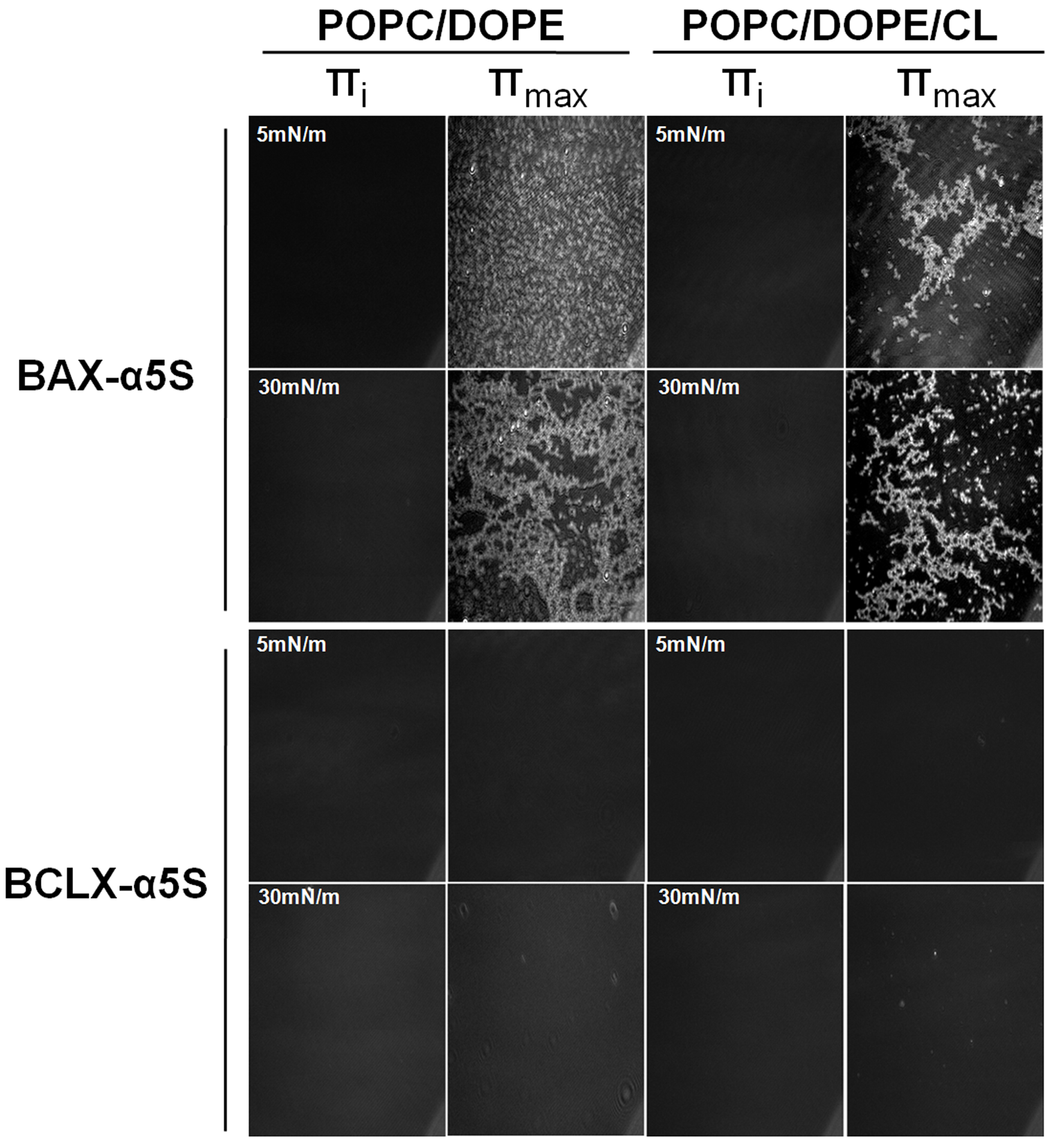

Supplement: Figure S3 — BAM images acquired at initial surface pressures of 5 mN/m or 30 mN/m for BAX-α5S and BCLX-α5S. The BAM images of MOM and MIM/MOM-like lipid monolayers were recorded before (right) and after (left) addition of 0.2 µM BAX-α5S or BCLX-α5S into the subphase. (10.20 MB TIF) [file pone.0009066.s003.tif]
